# Supplementary figures and images for: Characteristics of chemically induced liver progenitors derived from a pig model of metabolic dysfunction-associated steatotic liver disease
Source: PLoS One. 2024 Dec 5;19(12):e0313312. doi: 10.1371/journal.pone.0313312 (PMC11620392; doi:10.1371/journal.pone.0313312)

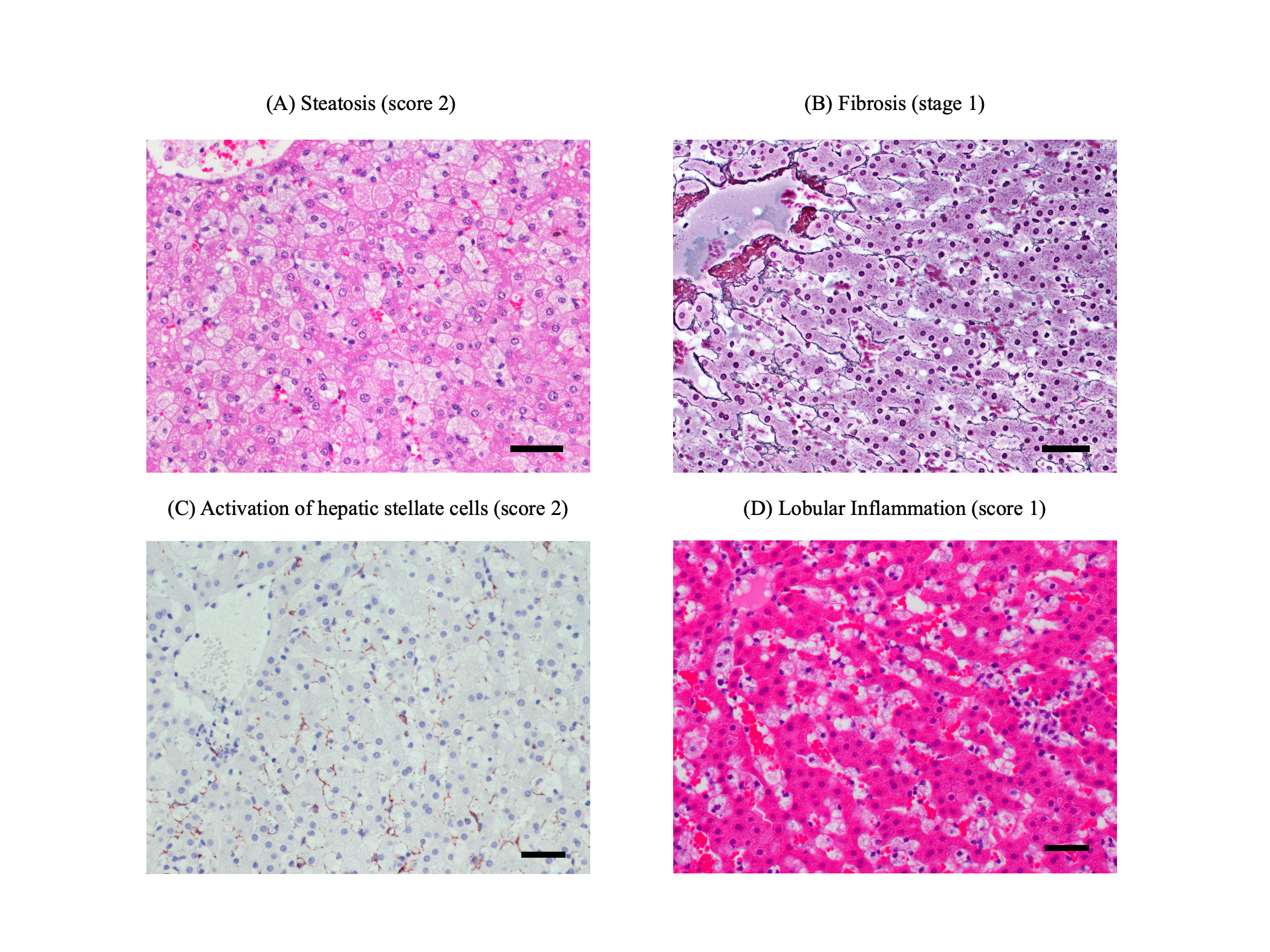

Supplement: S1 Fig — Steatosis score 2 (A), fibrosis stage 1 (B), activation of hepatic stellate cells score 2 (C), and lobular inflammation score 1 (D). MASLD, metabolic-dysfunction-associated steatotic liver disease. (TIF) [file pone.0313312.s001.tif]

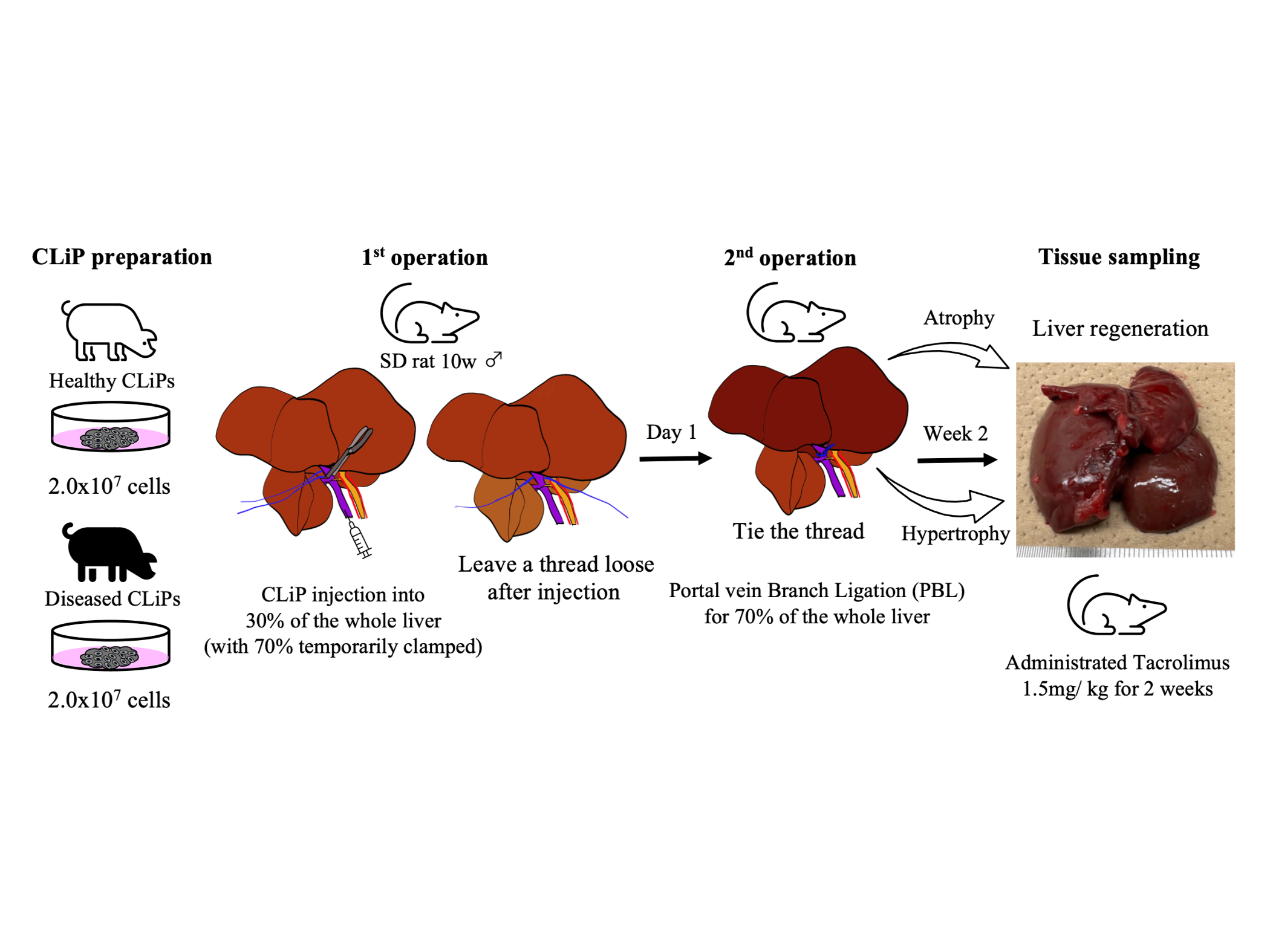

Supplement: S2 Fig — pCLiP, porcine chemically induced liver progenitors; PBL, portal vein branch ligation. (TIF) [file pone.0313312.s002.tif]

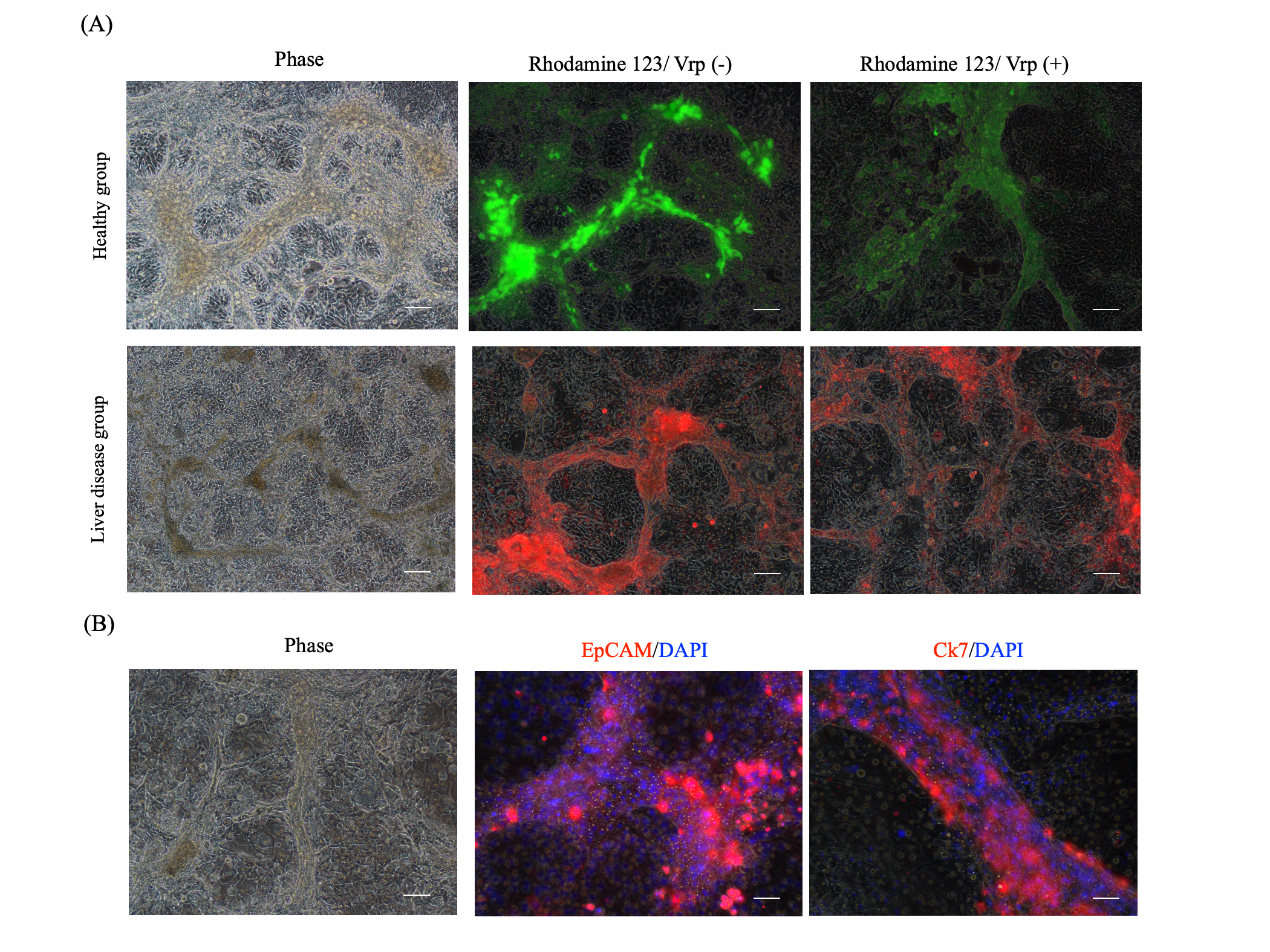

Supplement: S3 Fig — (A) Cell morphology after induction and evaluation of bile duct transporter activity with and without transporter inhibitor (verapamil [VRP]) using rhodamine 123 stainings (green and/or red). Bars represent 50 μm. (B) Immunofluorescence staining of the bile duct network structure of disease-derived pCLiP. Red: Complete ectoderm marker (EpCAM) or cholangiocyte marker (CK7); blue: Cell nuclei. Bars represent 50 μm. pCLiP, porcine chemically induced liver progenitors; EpCAM, epithelial cell adhesion molecule. (TIF) [file pone.0313312.s003.tif]
