# Supplementary material for: Characteristics of chemically induced liver progenitors derived from a pig model of metabolic dysfunction-associated steatotic liver disease
Source: PLoS One. 2024 Dec 5;19(12):e0313312. doi: 10.1371/journal.pone.0313312 (PMC11620392; doi:10.1371/journal.pone.0313312)
Supplement: S1 Table — (DOCX) [file pone.0313312.s004.docx]

**S1 Table. Normalized intensity values for microRNAs selected for clustering**

| Name | [H1] (normalized) | [H2] (normalized) | [D1] (normalized) | [D2] (normalized) |
| --- | --- | --- | --- | --- |
| hsa-miR-378b | -1.4591007 | -0.36935663 | -0.14663458 | -1.1040704 |
| hsa-miR-378a-3p | -1.0535381 | -0.35790586 | 0.22956848 | -0.12280035 |
| hsa-miR-214-3p | 0.48811722 | -0.55822563 | 0.15045023 | 0.6045346 |
| hsa-miR-21-5p | -0.886637 | -4.407571 | -0.2160945 | -4.4338365 |
