# Supplementary material for: Characteristics of chemically induced liver progenitors derived from a pig model of metabolic dysfunction-associated steatotic liver disease
Source: PLoS One. 2024 Dec 5;19(12):e0313312. doi: 10.1371/journal.pone.0313312 (PMC11620392; doi:10.1371/journal.pone.0313312)
Supplement: Attachment — Submitted filename: reivew.docx [file pone.0313312.s005.docx]

Title: Characteristics of chemically induced liver progenitors derived from a pig model of

metabolic dysfunction-associated steatotic liver disease

The authors reported that CLiP induced in the steatotic liver has the higher potential in the proliferative capacity than one in the healthy liver and the CLiP in both groups has the high cell viability ant the ability to differentiate into the albumin-positive cells. In addition, Exosome counts were lower in disease-derived CLiP than in the normal group; however, there were no differences in miRNA expression within exosomes. Finally, the authors mentioned that CLiP induced in the steatotic liver are a promising source for cell therapy in patients with liver disease. Although this study seemed very interesting, this manuscript needs some improvement in organization and a little more thought needs to be given to it. In addition, there are some questions in the manuscript. So, they should answer some question below:

Major revision

1: The authors explained that the mRNA levels of EVs secretory activity was proved. However, they did not described the protein level. Actually, I think that the authors need to check the protein level to establish if mRNA is working or not. Please add the explanation.

2: Extracellular vesicles can be broadly classified into three types based on their intracellular production mechanisms: exosomes, microvesicles, and apoptotic bodies. However, I felt that the authors used EVs and exosomes as if they are equivalent. I think the exosome and EVs experiments are important. Please add the explanation about them.

3: Why was the proliferative capacity in CLiP in the disease liver the higher that one in the normal liver? Liver disease itself already stimulated the capacity? Please add the explanation.

Minor revision

1: In the introduction, the authors explained MASLD in the last sentences. The order of the sentences seems odd; wouldn't MASLD's explanation be better at the beginning?

2: Why was CYP3A4 evaluated? The authors have not stated why it was evaluated, so please provide an explanation.

3: Why was CD133 positivity rate was measured in order to evaluate EpCAM and TROP2 in Flow cytometry? Please add the explanation.

4: Too many abbreviations and terms are used that are not listed in the Material and Methods. Please explain what they are used to evaluate, such as CD90, CD9, TROP2, PBLmodel, EpCAM, etc.

5: Regarding the immunostaining, the background is too irregular, especially in Figs. 1 and 3. I would like to see them changed to clearer pictures.
