# Supplementary material for: Characteristics of chemically induced liver progenitors derived from a pig model of metabolic dysfunction-associated steatotic liver disease
Source: PLoS One. 2024 Dec 5;19(12):e0313312. doi: 10.1371/journal.pone.0313312 (PMC11620392; doi:10.1371/journal.pone.0313312)
Supplement: Attachment — Submitted filename: reivew R1.docx [file pone.0313312.s007.docx]

Title: Characteristics of chemically induced liver progenitors derived from a pig model of

metabolic dysfunction-associated steatotic liver disease

The author has responded to the questions accurately, so I will accept the paper for this journal.
